# Prompt matters: evaluation of large language model chatbot responses related to Peyronie’s disease

**DOI:** 10.1093/sexmed/qfae055

**Published:** 2024-09-09

**Authors:** Christopher J Warren, Victoria S Edmonds, Nicolette G Payne, Sandeep Voletti, Sarah Y Wu, JennaKay Colquitt, Hossein Sadeghi-Nejad, Nahid Punjani

**Affiliations:** Department of Urology, Mayo Clinic Arizona, Phoenix, AZ 85054, United States; Department of Urology, Mayo Clinic Arizona, Phoenix, AZ 85054, United States; Department of Urology, Mayo Clinic Arizona, Phoenix, AZ 85054, United States; Mayo Clinic Alix School of Medicine, Scottsdale, AZ 85259, United States; Mayo Clinic Alix School of Medicine, Scottsdale, AZ 85259, United States; Mayo Clinic Alix School of Medicine, Scottsdale, AZ 85259, United States; Department of Urology, New York University, New York, NY 10016, United States; Department of Urology, Mayo Clinic Arizona, Phoenix, AZ 85054, United States

**Keywords:** large language model, Peyronie’s disease, chatbot, patient education, artificial intelligence

## Abstract

**Introduction:**

Despite direct access to clinicians through the electronic health record, patients are increasingly turning to the internet for information related to their health, especially with sensitive urologic conditions such as Peyronie’s disease (PD). Large language model (LLM) chatbots are a form of artificial intelligence that rely on user prompts to mimic conversation, and they have shown remarkable capabilities. The conversational nature of these chatbots has the potential to answer patient questions related to PD; however, the accuracy, comprehensiveness, and readability of these LLMs related to PD remain unknown.

**Aims:**

To assess the quality and readability of information generated from 4 LLMs with searches related to PD; to see if users could improve responses; and to assess the accuracy, completeness, and readability of responses to artificial preoperative patient questions sent through the electronic health record prior to undergoing PD surgery.

**Methods:**

The National Institutes of Health’s frequently asked questions related to PD were entered into 4 LLMs, unprompted and prompted. The responses were evaluated for overall quality by the previously validated DISCERN questionnaire. Accuracy and completeness of LLM responses to 11 presurgical patient messages were evaluated with previously accepted Likert scales. All evaluations were performed by 3 independent reviewers in October 2023, and all reviews were repeated in April 2024. Descriptive statistics and analysis were performed.

**Results:**

Without prompting, the quality of information was moderate across all LLMs but improved to high quality with prompting. LLMs were accurate and complete, with an average score of 5.5 of 6.0 (SD, 0.8) and 2.8 of 3.0 (SD, 0.4), respectively. The average Flesch-Kincaid reading level was grade 12.9 (SD, 2.1). Chatbots were unable to communicate at a grade 8 reading level when prompted, and their citations were appropriate only 42.5% of the time.

**Conclusion:**

LLMs may become a valuable tool for patient education for PD, but they currently rely on clinical context and appropriate prompting by humans to be useful. Unfortunately, their prerequisite reading level remains higher than that of the average patient, and their citations cannot be trusted. However, given their increasing uptake and accessibility, patients and physicians should be educated on how to interact with these LLMs to elicit the most appropriate responses. In the future, LLMs may reduce burnout by helping physicians respond to patient messages.

## Introduction

Patients with urologic concerns, such as those related to Peyronie’s disease (PD), resort to online resources to obtain information about their health.^1^ Prior research has demonstrated inaccurate and poor-quality information related to PD on the internet.^1^

Large language model (LLM) chatbots are an emerging platform for patients to obtain information about urologic conditions.^2^ They are a form of artificial intelligence that uses deep learning to excel at prompt-based interactions.^3^ They have demonstrated remarkable ability in various disciplines.^3^ Chatbots, however, are not without limitations and have been shown to create false information while sounding confident: a phenomenon known as *hallucination*.^3^

We examined the quality of information related to PD among various LLMs, and we explored opportunities for LLMs to reply to patient electronic health record (EHR) correspondence for PD surgery. To date, the majority of studies have focused on ChatGPT alone.^2^^,^^4^^,^^5^ We sought to utilize multiple chatbots to broaden our evaluation of their utility for clinicians and patients alike.

## Methods

### L‌LM selection

Three LLMs were chosen due to the popularity of their parent companies: ChatGPT, powered by GPT-3.5; Google Bard, powered by paLM2; and Bing AI, powered by GPT-4 (used in “balanced” mode). The last chatbot was chosen because it is marketed for health care providers: Doximity’s DocsGPT, powered by GPT-4.

### L‌LMs as a patient education resource

**Figure 1:**
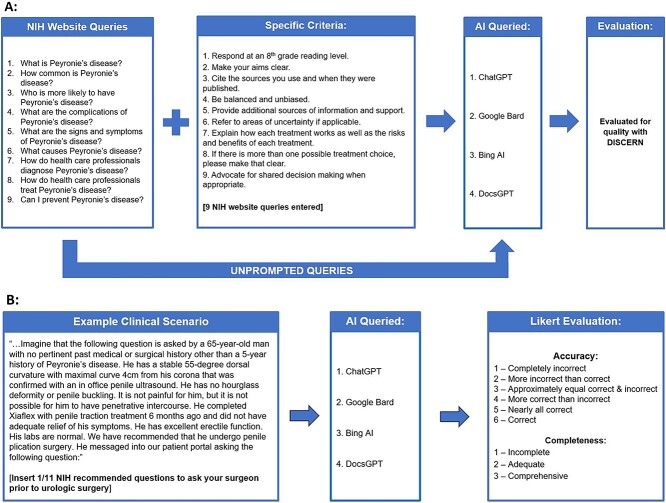
(A) Methodology for assessing the quality of information generated from prompted and unprompted queries related to Peyronie’s disease. (B) Methodology for evaluating chatbot responses to 11 questions recommended to ask a surgeon before undergoing urologic surgery, preceded by an abridged clinical scenario. AI, artificial intelligence; NIH, National Institutes of Health.

Frequently asked questions regarding PD from the National Institutes of Health (NIH) website were entered into each chatbot. A total of 9 questions were entered with use of a subsequent prompt to optimize the chatbot responses (Figure 1A). This was first completed in October 2023, and to ensure consistency among the chatbots, this was repeated in April 2024. Three postgraduate-level urology residents (C.J.W., V.S.E., N.P.), who were blinded to prompting status, evaluated these responses using the DISCERN criteria, a validated questionnaire designed for patients to assess the quality of written health care information.^6^ This Likert-type questionnaire is scored 1 to 5, with a score of 1 indicating “serious or extensive shortcomings” and a score of 5 reflecting “minimal shortcomings.” Consistent with previous literature, we considered a score <3 to be poor, 3 or 4 moderate, and >4 highly reliable.^7^

### Citation review

Each time that an LLM cited a source, 2 reviewers (J.C., S.Y.W.) verified its validity. Four scenarios were encountered: (1) The source was cited appropriately, and the information cited was contained in that source (confirmed source, contains information). (2) The source was real, but the information that was cited was not present in that source (confirmed source, hallucinated information). (3) The source cited was not where the hyperlink brought the user, but the information was in the linked source (hallucinated source, contains information). (4) The source was hallucinated, and the source did not contain the information (hallucinated source, hallucinated information).

### L‌LM performance in answering EHR correspondence related to PD

Each chatbot was prompted with a sample clinical scenario in a private web browser (Figure 1B). A prompted clinical scenario was used to simulate whether a chatbot with access to a patient’s medical record could answer preoperative patient questions related to PD surgery. This prompt was followed by a list of 11 questions recommended by the NIH that patients ask their physicians prior to undergoing urologic surgery.^8^ The previous conversation was deleted prior to each query. The same reviewers evaluated the accuracy and completeness of each question using a previously published Likert scale.^2–4^ A novel Likert scale was used to evaluate these questions because the DISCERN criteria are designed to evaluate patient education material (eg, patient handouts about PD) and thus would not be an appropriate tool to assess responses to specific surgical questions.

### Statistical analysis

After both rounds of evaluation were averaged, descriptive statistics were used to describe continuous variables, such as the DISCERN score, accuracy scale, and completeness scale. Differences between the prompted and unprompted searches were completed by a 2-tailed *t*-test. Statistical significance was measured at *P* < .05. Microsoft Excel was used to calculate statistics. Reading level was evaluated in Microsoft Word per the Flesch-Kincaid reading-level feature. Google Bard refused to answer 3 questions, and these values were excluded from analysis.

## Results

### L‌LMs as a patient education resource

Without prompting, the quality of information was moderate, with a mean 3.5 of 5.0 (SD, 0.6) across all LLMs. This improved with prompting, as the quality of information was rated high, with a mean DISCERN score of 4.6 of 5.0 (SD, 0.7; Table 1). Moreover, each chatbot performed at a level considered high quality when evaluating material as a patient education resource (>4.0/5.0).

**Table 1:** Quality of patient queries, prompted and unprompted, as evaluated by the DISCERN criteria.^a^

	**Unprompted query**		**Prompted query**	
**Language model**	**Quality (1-5)**	**Reading level**	**Quality (1-5)**	**Reading level**
ChatGPT	3.7 (0.5)	14.6 (1.8)	4.5 (0.8)	11.0 (1.2)
BARD	3.6 (0.5)	10.8 (2.1)	4.5 (0.6)	9.6 (1.4)
Bing AI	3.5 (0.6)	12.4 (1.8)	4.7 (0.5)	11.5 (1.5)
DocsGPT	3.3 (0.8)	13.8 (2.7)	4.6 (0.7)	9.9 (1.4)
Total	3.5 (0.6)^b^	12.9 (2.5)	4.6 (0.7)^b^	10.5 (1.6)

^a^Data are presented as mean (SD).

^b^Significant difference at *P* < .05.

### L‌LM performance in answering EHR correspondence related to PD

LLMs were accurate and complete, with average scores of 5.5 of 6.0 (SD, 0.8) and 2.8 of 3.0 (SD, 0.4), respectively (Table 2). Each chatbot was highly accurate and complete, with no chatbot average <5.4 (SD, 0.9) or <2.8 (SD, 0.4) when answering questions. However, Google Bard refused to answer questions 3, 8, and 10, citing that it was not a health care professional and not qualified to give medical advice.

**Table 2:** Accuracy and completeness of large language model responses to questions from a hypothetical patient with Peyronie’s disease who was recommended penile plication surgery.^a^

	**Answer to simulated patient questions, mean (SD)**	
**Language model**	**Accuracy (1-6)**	**Completeness (1-3)**
ChatGPT	5.4 (0.9)	2.8 (0.4)
BARD	5.7 (0.6)	2.9 (0.3)
Bing AI	5.4 (0.6)	2.9 (0.4)
DocsGPT	5.7 (0.7)	2.9 (0.2)
Total	5.5 (0.8)	2.8 (0.4)

^a^For full clinical scenario, see Appendix A1.

### Reading level

The average Flesch-Kincaid reading level was high, with a mean grade level of 12.9 (SD, 2.5; Table 1). Chatbots were unable to communicate at a grade 8 reading level, even when prompted to do so, responding at grade 10.5 (SD, 1.6). In contrast, the reading level of the NIH website is lower, with a mean reading level of 9.8 (SD, 2.1).

### Citation review

In total, there were 254 cited sources in our prompted cohort (Appendix A2); of those, 108 were appropriately cited. However, in 101 (39.8%) cases, the cited source was not real, and the information that it claimed was in the citation was not found. There were 28 (11.0%) instances where the cited source was real but the information that it cited was not present in that source. Finally, in 16 (6.3%) citations, the linked source directed the user to a different source where the information was contained.

## Discussion

The current study demonstrates that artificial intelligence chatbots can provide high-quality information to patients about PD, but the quality of information generated is dependent on the user’s prompts. Moreover, given a clinical scenario, LLMs were able to accurately and comprehensively answer preoperative questions that the NIH recommends patients ask their surgeon prior to urologic surgery. However, the citations provided by chatbots cannot be trusted, with appropriate citations being found only 42% of the time. In addition, the average reading level of their responses is higher than that of the average American.

Except for 3 questions that Google Bard refused to answer, we found that all LLMs studied answered preoperative patient questions with high accuracy and provided complete answers. This builds on the work of Goodman et al, who queried ChatGPT with physician-generated questions across 17 specialties to assess the accuracy and completeness of each question.^3^ Using the same Likert scale, they found that ChatGPT was able to provide information that was accurate (median, 5.5/6.0) and complete (median, 3.0/3.0) with no differences observed in difficulty level. In contrast to their work that demonstrated the accuracy of physician-generated questions, we showed the accuracy of patient-generated questions, with implications for implementation as a patient education resource that may lower the administrative burden on urologists.

Our findings, in combination with previously published work, demonstrate that chatbot output quality is dependent on user prompting. This was shown by Coskun et al, who queried ChatGPT with 59 queries similar to Google searches from the European Association of Urology website.^4^ They found that the information was not high quality, averaging 3.62 of 5 (SD, 0.49). Our unprompted queries were similar with their results, but our prompted queries were higher. Moreover, Manolitsis et al showed that ChatGPT can be reprogrammed to answer questions related to urinary tract infections in a more appropriate manner than the standard version, whereas we found that the current version can be prompted to answer more accurately simply by asking the question in a specific way.^9^ ChatGPT, with its open source code, is highly customizable. It is feasible that a custom chatbot could be created that is preprompted, PD specific, and given strict reading-level instructions that can be utilized by patients and physicians alike as an educational resource.

Reading level did not improve with prompting. All chatbots queried answered the unprompted queries at a reading level of 12.9 (SD, 2.5), which is above the recommended reading level of grade 8.^1^ Even when prompted to do so, they could not lower their reading levels appropriately, with a mean grade of 10.5 (SD, 1.6). These findings add to work from Davis et al, who found that ChatGPT was able to respond appropriately to 18 of the most searched questions related to urology using Google Trends but that it responded at a high average Flesch-Kincaid reading level at 13.5 (SD, 1.74).^2^

We found a concerning number of hallucinations related to citations. The examined chatbots appropriately cited sources in only 108 of 254 citations (42.5%). Disturbingly, the chatbots hallucinated sources almost as frequently as they cited them appropriately (101/254, 39.8%). This is consistent with work from Whiles et al, who found that ChatGPT hallucinated 12 of 13 citations.^10^ Although the responses are accurate and complete, patients and physicians must be advised that they cannot trust the sources provided by chatbots.

Our study is not without limitations. First, any study related to chatbots is inherently flawed given their ability to generate a unique answer each time that a question is asked and their constant improvement. We overcame this by asking and evaluating each question at 2 distinct time points separated by several months. Second, we used a Likert scale for accuracy that is not validated in this context but has been used in similar studies.^3^ Last, our reviewers were postgraduate trainees and not board-certified urologists. While the DISCERN questionnaire is designed to be used by patients with no medical training and thus does not require a board-certified urologist, the Likert scale for accuracy may be answered differently by more experienced urologists.

## Conclusion

LLM chatbots have the potential to be a valuable tool for patient education as well as answering patient messages in the EHR. However, they are limited by their prerequisite reading level, and their citations cannot be trusted. Patients and physicians must be educated on how to interact with these chatbots to elicit appropriate responses, and they should be advised to use caution when sources are cited.

## Supplementary Material

PD_AI_Project_Appendix_1_2_qfae055
