# Supplementary material for: Prompt matters: evaluation of large language model chatbot responses related to Peyronie’s disease
Source: Sex Med. 2024 Sep 9;12(4):qfae055. doi: 10.1093/sexmed/qfae055 (PMC11384107; doi:10.1093/sexmed/qfae055)
Supplement: PD_AI_Project_Appendix_1_2_qfae055 [file pd_ai_project_appendix_1_2_qfae055.docx]

**Appendix 1: Example clinical scenario, simulated patient question, and Large Language Model Chatbot Response:**

**Clinical Scenario**: Hi, this is a thought experiment to see if you could decrease administrative burden on urologists. Please answer the question in your prompt to the best of your ability. In your answer, please do not ask the patient to speak with his urologist or any other medical staff. Assume that all the risks and benefits of surgery have already been discussed. Try to use an 8th grade reading level when possible. Imagine that the following question is asked by a 65-year-old man with no pertinent past medical or surgical history other than a 5-year history of peyronie’s disease. He has a stable 55-degree dorsal curvature with maximal curve 4cm from his corona that was confirmed with an in office penile ultrasound. He has no hourglass deformity or penile buckling. It is not painful for him, but it is not possible for him to have penetrative intercourse. He completed Xiaflex with penile traction treatment 6 months ago and did not have adequate relief of his symptoms. He has excellent erectile function. His labs are normal. We have recommended that he undergo penile plication surgery. He messaged into our patient portal asking the following question:

**Simulated Patient Question:** What are the different types of surgery that I can have?

**Docs GPT answer:** Hello, For Peyronie's disease, there are a few different types of surgery that can be considered.

1. Penile Plication: This is the surgery we have recommended for you. In this procedure, the surgeon makes small stitches, or plications, on the side of the penis opposite the curve. This can straighten the penis. The benefit of this surgery is that it's less likely to affect erectile function.

2. Incision or Excision and Grafting: In this procedure, the surgeon makes a cut (incision) or removes (excises) part of the plaque in the penis that is causing the curve, and then patches the area with a graft. This surgery is usually considered for men with more severe curvature or deformity. However, it has a higher risk of causing issues with erectile function.

3. Penile Implants: This is usually considered as a last resort for men who also have erectile dysfunction. The surgeon inserts a device that makes the penis firm enough for intercourse. This surgery can straighten the penis and provide a solution for erectile dysfunction, but it's the most invasive option.

Each of these surgeries has its own benefits and risks, and the best choice depends on the specifics of your condition and your personal preferences.

**Appendix 2: Validity of citations stratified by Large Language Model (LLM)**

| **LLM** | **Total # Citations** | **Confirmed source, Contains information: n (%)** | **Confirmed source, hallucinated information: n (%)** | **Hallucinated source, contains information: n (%)** | **Hallucinated source, hallucinated information: n (%)** |
| --- | --- | --- | --- | --- | --- |
| Bard | 69 | 34 (49.3%) | 11 (15.9%) | 9 (13.0%) | 14 (20.3%) |
| Bing | 61 | 39 (63.9%) | 4 (6.6%) | 7 (11.5%) | 11 (18.0%) |
| ChatGPT | 62 | 10 (16.1%) | 7 (11.3%) | 0 | 45 (72.6%) |
| DocsGPT | 62 | 25 (40.3%) | 6 (9.7%) | 0 | 31 (50.0%) |
| **TOTAL** | **254** | **108 (42.5%)** | **28 (11.0%)** | **16 (6.3%)** | **101 (39.8%)** |
